# Removal or retention of minimally invasive screws in thoracolumbar fractures? Systematic review and case–control study

**DOI:** 10.1007/s00701-023-05514-9

**Published:** 2023-02-15

**Authors:** Ravindran Visagan, Siobhan Kearney, Sebastian Trifoi, Nida Kalyal, Florence Hogg, Beatrice Quercetti, Mohamed Abdalla, Mihai Danciut, Marios C. Papadopoulos

**Affiliations:** Department of Neurosurgery, St. George’s Hospital, Atkinson Morley Wing, London, SW17 0QT UK

**Keywords:** Spinal fracture, Pedicle screw, Fixation, Outcome

## Abstract

**Background:**

There is uncertainty regarding delayed removal versus retention of minimally invasive screws following percutaneous fixation for thoracolumbar fractures. We conducted a systematic review and case–control study to test the hypothesis that delayed metalwork removal following percutaneous fixation for thoracolumbar fractures improves outcome.

**Methods:**

A systematic review was performed in accordance with the PRISMA guidelines. Our case–control study retrospectively evaluated 55 consecutive patients with thoracolumbar fractures who underwent percutaneous fixation in a single unit: 19 with metalwork retained (controls) and 36 with metalwork removed. Outcomes were the Oswestry Disability Index (ODI), a supplemental questionnaire, and complications.

**Results:**

The systematic review evaluated nine articles. Back pain was reduced in most patients after metalwork removal. One study found no difference in the ODI after versus before metalwork removal, whereas three studies reported significant improvement. Six studies noted no significant alterations in radiological markers of stability after metalwork removal. Mean complication rate was 1.7% (0–6.7). Complications were superficial wound infection, screw breakage at the time of removal, pull-out screw, and a broken rod. In the case–control study, both groups were well matched. For metalwork removal, mean operative time was 69.5 min (range 30–120) and length of stay was 1.3 days (0–4). After metalwork removal, 24 (68.6%) patients felt better, 10 (28.6%) the same and one felt worse. Two patients had superficial hematomas, one had a superficial wound infection, and none required re-operation. Metalwork removal was a significant predictor of return to work or baseline household duties (odds ratio 5.0 [1.4–18.9]). The ODI was not different between groups.

**Conclusions:**

The findings of both the systematic review and our case–control study suggest that removal of metalwork following percutaneous fixation of thoracolumbar fractures is safe and is associated with improved outcome in most patients.

**Supplementary Information:**

The online version contains supplementary material available at 10.1007/s00701-023-05514-9.

## Introduction

Approximately 90% of spinal fractures are thoracolumbar [7]; some of which are managed conservatively [1, 7] and some operatively by pedicle screws and rod fixation [3, 18, 23]. Increasingly, surgical fixation is performed percutaneously rather than open [3, 18, 23]. Advantages of the percutaneous versus the open technique include: smaller wounds, less blood loss, shorter surgery, less pain, less muscle dissection, shorter stay, and lower infection rate [18].

Once the fractures have healed, some surgeons remove the metalwork while others leave it in situ [24]. A meta-analysis of the removal versus retention of metalwork after open placement found variable outcomes with sample and study heterogeneity; removal in younger patients achieved superior functional outcomes without progressive deformity [12]. The argument for removing the metalwork is more compelling for percutaneous than open surgery, because removing percutaneously placed screws and rods is a small procedure that involves re-opening the small stab incisions and little tissue dissection compared with re-opening the long midline incision and extensive muscle dissection when removing openly placed pedicle screws and rods [15].

To investigate the evidence base in favour of removal versus retention of percutaneously placed metalwork, we performed a systematic review of relevant studies. This identified a gap in knowledge regarding which management option is superior. In our unit, some spinal surgeons do and some do not routinely remove the metalwork after percutaneous placement in neurologically intact patients. We exploited this variability in practice, performing a case–control study, to evaluate whether the routine removal of metalwork in these patients is beneficial. Based on published evidence and our study, we analyse whether the two are congruent and propose guidelines for the routine removal of metalwork after percutaneous pedicle screw and rod fixation for thoracolumbar fractures.

The specific aims of the study were.

1. To perform a systematic review to evaluate if there are clinical and/or functional differences in patients’ in whom metalwork is removed
2. To test the hypothesis that following percutaneous fixation for thoracolumbar fractures, metalwork removal improves functional outcome

## Materials and methods

### Systematic review

#### PICO

Population: patients with thoracolumbar fractures who underwent percutaneous pedicle screw/rod fixation.

Intervention: elective removal of metalwork.

Comparison: retention of metalwork.

Outcome: any clinical or functional outcome.

#### Systematic search

A systematic search according to PRISMA guidelines utilizing the PubMed, EMBASE, Cochrane Database and Google Scholar was conducted. The databases were queried with the following search terms using different Boolean combinations to maximize data capture: ‘percutaneous’, ‘implant’, ‘instrument*’, ‘fixation’, ‘metalwork’, ‘metalware’, ‘remov*’, ‘thoracolumbar’, ‘vertebra’, and ‘fracture’. Only studies with the full text available in English were included. All retrieved titles and abstracts were independently screened by two reviewers (RV and MCP). The full texts of the most relevant studies were evaluated to assess their eligibility for inclusion.

#### Inclusion/exclusion

Inclusion criteria were 1) randomized or non-randomized controlled trials, cohort, or case series examining thoracolumbar fractures with or without neurological deficit, 2) posterior percutaneous fixation of thoracolumbar fractures with planned removal of metalwork, and 3) reported clinical or radiological follow-up outcomes after metalwork removal, relating to pain and/or functional status. The included studies were stratified according to the presence or absence of neurological deficit.

### Case control study

#### Patients

We performed a retrospective service evaluation of patients consecutively treated in our unit (2014–2020). The inclusion criteria were: 1) thoracic or lumbar fracture, 2) traumatic injury, and 3) percutaneous pedicle screw or rod fixation. The exclusion criteria were 1) neurological deficit at presentation, 2) non-traumatic cause (e.g., cancer), 3) open pedicle screw/rod fixation, and 4) no follow-up. The patients were divided into two groups according to whether the metalwork was retained (group A) or removed (group B).

#### Baseline characteristics

For all patients, we collected demographics (age at injury, sex, and Charlson morbidity index), and injury characteristics (AO classification, mechanism, other injuries, and levels). There are different methods of accounting for the confounding effect of multiple variables. We opted to perform univariate logistic regression and then include all significant variables in a multivariate logistic model.

#### Metalwork insertion

Percutaneous pedicle screw fixation was performed under general anaesthesia with the Stryker ES-II system using bi-planar X-ray guidance. The operating surgeon determined the number of vertebral levels to be fixed. We recorded the number of fixed levels (i.e., L2–4 fixation for L3 burst fracture denotes a 2-level fixation reflecting the number of instrumented vertebral levels), duration of surgery, blood loss, length of stay (LOS), and complications.

#### Metalwork removal

Metalwork removal surgery was performed 1 year (median; range 5–27 months) post-fixation. The decision for removal at this time was dependent upon patients’ symptoms, satisfactory post-operative imaging, and patient/surgeon preference. Surgery was performed under general anaesthesia by re-opening the stab incisions to remove the blockers, rods, and screws. We recorded the duration of surgery, blood loss, LOS, and complications.

##### Long-term follow-up

For all patients, we recorded the post-operative Oswestry Disability Index (ODI) [5] and a 6-point questionnaire administered by phone or post as follows:

1. If you still take pain medication, please write down the names of the medications and how often you take them.
2. What date was your original operation?
3. Have you had your metalwork removed? If so, what date was it done?
4. Did you require any further spinal operations after removal of your metalwork?
5. Did removing the metalwork make you feel better, worse, or no change?
6. Have you returned to work or homemaking duties? If not, can you state why?

### Statistical analysis

Data are presented as mean (range) for continuous variables and frequency (%) for categorical variables. Baseline characteristics for group A versus group B were compared using the Student’s *t*-test (Microsoft Excel for Mac 2021, v.16.54) or Fisher’s exact test. Statistical analyses were performed using XLSTAT-Life Sciences software (v. 2021.5 by Addinsoft). Binary logistic regression was used to identify predictive factors for: 1) return to work or baseline activities and 2) analgesia use. Multinomial logistic regression was used to identify the predictive factors for the ODI. Statistical significance was set at *P* < 0.05.

## Results

Initially, we present the preliminary findings of the (i) systematic review and (ii) case–control study. Then, we present a parameter-based comparative analysis of the findings between the systematic review then our-case control study, and summaries. Parameters evaluated include timing of metalwork removal, pain outcomes, disability, return-to-work status, deformity, and complication rates.

### Systematic review

#### Study inclusion

**Fig. 1:**
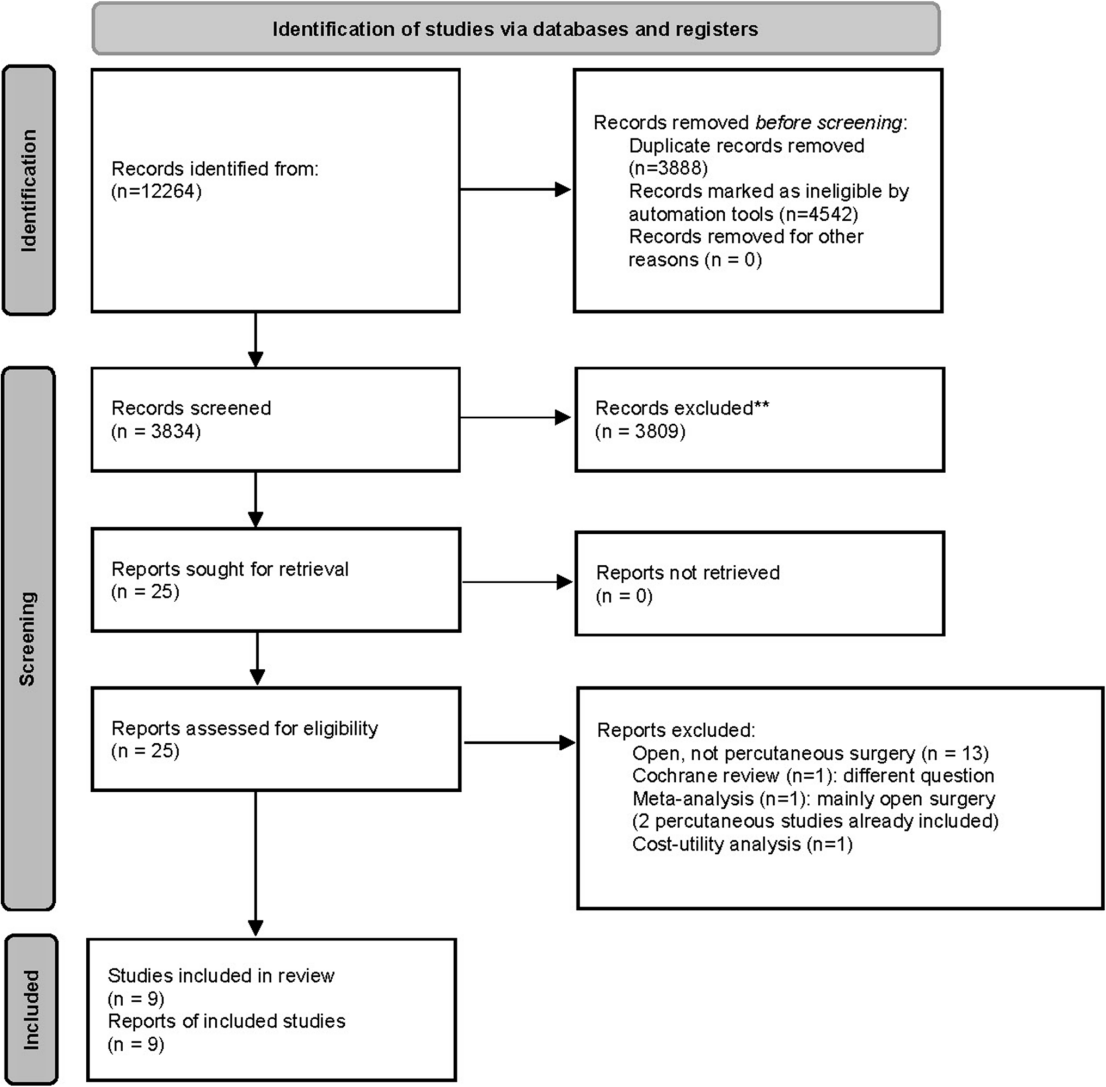
PRISMA 2020 flow diagram for systematic review

A systematic search yielded 12,264 articles, which were reduced to 8,376 after removing duplicates; 4,542 articles were removed using automation tools, leaving 3,834 abstracts for screening. After excluding papers not relevant to our PICO query, there were 25 left for full-text eligibility assessment, of which 9 were ultimately included in the review [2, 4, 8, 11, 13, 14, 16, 20, 21] (Fig. 1) [17]. Reasons for exclusion were other fracture locations, non-percutaneous/open or heterogeneous fixation techniques, unreported parameters of interest, or whether the study research question was irrelevant to our PICO query. Table 1 summarizes the nine relevant studies. There are two class II (prospective), six class III (retrospective), and one class IV (case series) studies. Six studies consider neurologically intact patients and three consider patients with a neurological deficit. The one study comparing patients with metalwork removed versus not removed [14] had selection bias as the metalwork removed group comprises 6 cases with complications from the primary surgery that prompted the metalwork removal. No other study other than our own compares instrumentation removed versus retained patient groups. Therefore, from the systematic review, there are only comparisons of pre- versus post-metalwork removal. No studies comprehensively looked at postoperative analgesia requirement, return to work/functional status, and operative factors (blood loss, operative time, and length of stay) of the metalwork removal group. These were incorporated in our case–control study.

**Table 1:** Baseline characteristics

Characteristic	Description	Group A: metalwork not removed	Group B: metalwork removed	*P* value
Patients	Number	19	36	
Age	Years (mean, range)	45.9, 23–75	42.1, 12–67	0.363 (Fisher exact)
Sex	Male:female (%)	13:6 (68.4:31.6)	15:21 (41.7:58.3)	0.089 (Fisher exact)
Charlson index	Mean, (range)	0.51 (0–3)	0.84 (0–3)	0.245 (Fisher exact)
Injury level	Number of patients T, TLJ, L (%)	2, 12, 5 (10.5, 63.2, 26.3)	5, 21, 10 (13.9, 58.3, 27.8)	0.960 (Fisher exact)
AO classification	Number of patients A, B, C (%)	16, 2, 1 (84.2, 10.5, 5.3)	22, 12, 2 (61.1, 33.3, 5.6)	0.142 (Fisher exact)
Mechanism of injury	Number of patients Fall < 2 m, fall > 2 m, RTA, other (%)	3, 10, 6, 0	7, 18, 9, 2	0.862 (Fisher exact)
Vertebrae fixed	2, 3, 4/number of patients (%)	12, 2, 5	14, 3, 19	0.185 (Fisher exact)
Spinal fixation	Litres blood loss < 0.1, 0.1, 0.2, 0.3 (%)	13, 0, 4, 2 (68.4, 0.0, 21.1, 10.5)	25, 1, 9, 1 (69.4, 2.8, 25.0, 2.8)	0.710 (Fisher exact)
	Mean (range) days length of stay	8.4 (3–19)	7.9 (3–20)	0.716 (t-test)
Follow-up from injury	Mean (range) months	44.9 (10–83)	43.3 (11–76)	0.807 (t-test)

*C.I.*, confidence interval; *L*, lumbar; *RTA*, road traffic accident; *T*, thoracic; *TLJ*, thoraco-lumbar junction

### Case control study

#### Patient characteristics

One hundred patient records met the inclusion/exclusion criteria, of which complete follow-up was available for 55 cases; all of which were included. Overall, mean age was 43.4 years (range 12–74), male:female was 1.04:1 (50.9%:49.1%), 7 fractures were only thoracic, 15 only lumbar, and 33 thoracolumbar, mean number of levels fixed was 3 (2–4), minimal blood loss (< 100 ml) was commonest (67.3%; in the remainder, mean was 205 mL [100–300]), and mean length of stay was 8.1 days (3–20). We then compared the 19 group A versus the 36 group B patients. There was a higher proportion of females in group B but this difference was not significant. Patients were followed-up for a mean of 44.9 months (10–83) in group A and 43.3 months (11–76) in group B post-surgery. Table 1 shows no significant differences in group A versus group B patients for demographics, co-morbidities, level of injury, fracture morphology, injury mechanism, operative levels fixed, number of screws placed, blood loss, and complications.

### Comparative analysis of parameters between systematic review and case–control study

#### Timing of metalwork removal

The timing of metalwork removal surgery was clearly reported in 7/9 (77.8%) studies. Overall, the mean was 14.1 months (10–24), compared with 13.3 (5–27) months in our study (Table 2).

**Table 2:** Systematic review evaluating metalwork removal after percutaneous fixation for thoracolumbar fractures

Neurologically intact				
Study	Class of evidence	Population and surgery	Outcomes	Summary/conclusions
Lorente et al. 2021^10^	II—prospective multicentre cohort	*n* = 31. All T11-L5 type A, AO classification fractures. Metalwork removal after 24 months	VAS, ODI, CL, FA, SI, C%, DD, DA	• Metalwork removal safe in type A fractures, good clinical results, no loss of correction • VAS, ODI improved post-removal but not significant • Post-removal: CL, FA, SI, kyphosis, C%, DD, DA not significantly different • 2 superficial wound infections (oral antibiotics, no sequelae)
Sasagawa et al. 2021^11^	III—retrospective series	*n* = 24. 2 groups of *n* = 12 (+ residual back pain, no residual back pain). Metalwork removal: mean 14.4 months (5 – 27) post index surgery	Patient satisfaction, NRS-B/L, ODI, local kyphosis, disc degeneration	• 13 extremely satisfied, 8 moderately satisfied, 3 neither, 0 dissatisfied • Patient satisfaction post removal high independent of residual back pain or occupation • No difference in disc degeneration or local kyphosis in patients with/without back pain • No complications reported
Manson et al. 2020^12^	II—prospective study	*n* = 32. (In 26, metalwork retained; in 6, metalwork removed). *n* = 26 neurologically intact (N0); *n* = 6 had deficit (1 N1, 2 N2, 3 N3). Removal at 16–45 months post insertion	Operative time, blood loss, LOS, patient satisfaction, NRS-B/L, ODI, RTW	• 26 (81.25%) with metalwork retention: minimal disability, mild pain, satisfaction and RTW (mean 6 months) with moderate back and leg pain until removal • 6 had removal of metalwork due to prominence of construct / screw loosening causing symptoms (removal at 16–45 months). RTW mean 7 months • After removal, minimal disability and mild pain reported • No complications after metalwork removal
Oh and Seo 2019^13^	III—retrospective series	*n* = 30. TLICS > 4. All neurologically intact. Metalwork removal at mean 12.8 months post index surgery	Optimal removal time assessed using: VAS, ODI, ROM, CA, AVHR, complications	• Metalwork removal group within 12 months had better ROM recovery than those removed after 12 months • No significant difference in VAS, ODI, AVHR or CA between groups • 2 cases of screw breakage—satisfactory outcomes at last follow-up
Kim et al. 2014^14^	III—retrospective series	*n* = 16 undergoing metalwork removal at 12 months. All neurologically intact. Two groups (A, B) depending on presence of osteoporosis. Metalwork removal at 12 months post index surgery	VAS, VH, ROM	• Significant pain relief in both groups • Significant improvement in VH post-op despite some VH post-removal • ROM improved significantly pre-removal and at last follow-up post-removal (10.5° and 10.2° in groups A and B at last follow-up) • Removal of metalwork preserves motion regardless of osteoporosis • No major complications from metalwork removal
Cox et al. 2013^15^	IV—case series	*n* = 3 with severe pain refractory to analgesia/bracing following percutaneous fracture fixation	Pain relief, radiographic fusion, complication rate	• All had significant pain reduction facilitating rapid mobilization • Radiographic fracture healing at 6 months • No complications
With neurological deficit				
Study	Class of evidence	Population and surgery	Outcomes	Summary/conclusions
Han et al. 2021^16^	III—retrospective series	*n* = 31. AO Neurologic status: 18 (58%) N0, 1 (3%) N1, 3 (10%) N2, 9 (29%) N3. Metalwork removal at median 13.7 months (6–56) post index surgery	CA, ODI, VAS, VBH, SMA	• Back pain relieved and ODI improved post metalwork removal (*p* < 0.001) irrespective of radiological outcomes • Less CA (1.58°), VBH (0.52 mm) • SMA preservation in 58.1%—significant if screw removal < 12 months • Kyphotic recurrence in 12.9%; but pain improved, no further surgery
Chen et al. 2020^17^	III—retrospective review	*n* = 84 with neurologic deficit. Decompression + intracorporeal bone grafting (minimal-access tubular-assisted) + percutaneous short segment pedicle screw fixation. Metalwork removal at 12 months post index surgery	ASIA, VAS, ODI, CSI, KA, CA, AVH, SI	• AIS: 14 (16.7%) did not improve 33 (39.3%) recovered to grade E. 5/9 AIS B improved by ≥ 1 grade. 32/44 (72.7%) recovered by 1 grade, 4 reached grade E. All grade D recovered to E At final follow-up post metalwork removal: • VAS and ODI decreased • Incidence of kyphosis recurrence limited—VBH and SI maintained • One pull-out screw, one broken rod
Proietti et al. 2019^18^	III—retrospective review	*n* = 36. Metalwork removal at mean 10 months	SF-12, ODI VAS, loss of correction, residual mobility (Dvorak)	• Clinically significant decrease in VAS + ODI at 1-month post-removal • Preserved mobility of treated segments post-removal at 12 months • Non-fused segment included in pedicle instrumentation maintains mobility if metalwork removed < 10 months • Post-removal in 12 months, normal ROM restored in proximal and distal segment of fracture, no significant loss of correction • No complications reported

*AO spine*, spine trauma classification modifiers: neurologically intact (N0); *N1*, transient neurologic deficit; *N2*, nerve root injury; *N3*, incomplete spinal cord injury or incomplete/complete cauda equina injury; *N4*, complete spinal cord injury; *AIS*, American spinal injury association Impairment Scale; *AVHR*, anterior vertebral height ratio; *CA*, Cobb’s angle; *CL*, correction loss; *CSI*, canal stenosis index; *C%*, compression %; *DA*, deformation angle; *DD*, degree of displacement; *FA*, fracture angle; *KA*, correction loss; *LOS*, length of stay; *NRS-B/L*, numeric rating scales for back and leg pain; *ODI*, Oswestry disability index; *ROM*, range of movements; *RTW*, return to work; *SI*, sagittal index; *SF-12*, 12-item short form survey; *SMA*, segmental motion angle; *TLICS*, Thoracolumbar Injury Classification and Severity Scale; *VAS*, Visual Analog Scale; *VBH*, vertebral body height; *VH*, vertebral height loss

*Summary*: timing of metalwork removal was similar in the review and our study.

### Pain outcomes

Six (66.7%) out of 9 studies [2, 8, 11, 13, 14, 20] used the visual analogue score and reported less pain after versus before metalwork removal; 1/9 (11.1%) study [14] concluded minimal pain post-metalwork removal; 1/9 (11.1%) study [16] reported no difference in pain between patients who had the metalwork removed before versus after 12 months of insertion. In our study, the pain component of the ODI and supplemental questionnaire captured pain evaluation. Most patients reported metalwork removal as beneficial: 24 (68.6%) felt better, 10 (28.6%) the same, and 1 worse (2.8%). Our univariate binary logistic regression model found that the number of fixed levels was a predictor of analgesic use, i.e., the greater the number of levels fixed, the increased likelihood of long-term analgesia requirement. This finding remained significant in the multivariate analysis (Supplemental Table 1).

*Summary*: pain outcomes are more frequently improved post metalwork removal in the review and our study.

### Disability

One (11.1%) out of nine studies found no difference in ODI after versus before metalwork removal [16], whereas 3/9 (33.3%) studies reported significant improvement in ODI [2, 8, 20], all in patients with preoperative neurological deficit. In our study, the mean ODI at follow-up was lower in group B than in group A, but the difference was not significant. A multinomial logistic regression model revealed that metalwork removal, age, Charlson co-morbidity index, spinal injury level, and number of levels fixed were not significant predictors of ODI (Supplemental Table 2).

*Summary*: Although the systematic review showed that ODI tends to be better in post-metalwork removal, our study did not replicate this finding to statistical significance.

### Return to work

Only 1/9 (11.1%) studies examined return to work [14], reporting mean of 7 months in the metalwork removed group versus 6 months in the metalwork retained group (not significant); in this study, the metalwork was removed due to complications from the primary surgery. In our study, a univariate binary logistic regression model showed that group B patients are 5 times more likely to return to work or baseline household activities than group A patients (Table 3). Age, Charlson co-morbidity index, injury level, and the number of fixed levels were not significant predictors of return to work or baseline household activities.

**Table 3:** Binary logistic regression. Dependent variable is return to work or return to household duties (Yes = 1, No = 0)

Predictor	Values	Odds ratio	95% C.I	*P* value
*Univariate*				
Screws removed	Yes = 1, No = 0	5.000	1.399–18.868	0.013^*^
Age	Years	0.999	0.959–1.041	0.969
Charlson	Comorbidity	0.658	0.361–1.198	0.171
Injury level	T = 1, TLJ = 2, L = 3	0.880	0.326–2.377	0.801
Vertebra	Number of levels	0.829	0.442–1.554	0.559

*C.I.*, confidence interval; *L*, lumbar; *T*, thoracic; *TLJ*, thoraco-lumbar junction

^*^Significant difference

*Summary*: Our finding of enhanced return to work/function, unaffected by several other variables, is not consistent with the single study from the review that evaluated this parameter. Clearly, this is an understudied yet critical metric, which warrants further evaluation.

### Deformity

Seven (77.8%) out of 9 studies examined various radiographic markers of stability/deformity (correction loss, fracture angle, sagittal index, compression %, degree of displacement, deformation angle, local kyphosis, Cobb angle, disc degeneration, vertebral height loss, range of motion, radiographic fusion, segmental motion angle, canal stenosis index, and residual mobility). Of these seven studies, six (85.8%) concluded that there was no significant alteration in markers of stability after versus before metalwork removal. Although 1/7 (14.2%) studies [8] noted kyphotic recurrence in 4/31 (12.9%) patients after metalwork removal, there were significant improvements in pain measures in these patients and no patient required additional surgery. Our study did not specifically examine radiological markers of stability. No patients from our removal group required further surgery for progressive instability.

*Summary*: in the vast majority of cases from the review, there is no clinically significant progressive deformity warranting further surgery following metalwork removal.

### Complications

Reported risks of metalwork removal surgery from the review were low with a mean complication rate of 1.7% (0–6.7). Complications were minor, including superficial wound infection, screw breakage at the time of removal, one pull-out screw, and broken rod. No studies specifically examined blood loss, operative time, and length of stay, which we examined in our study. In our study, metalwork removal surgery had minimal blood loss in 94% of cases, mean operative time was 69.5 min (39–120), and mean length of stay was 1.3 days (0–4). Complication rate was low (*n* = 3/33, 9.1%) including 2 superficial hematomas and 1 superficial wound infection; all were managed conservatively with no long-term adverse sequelae (Table 4). Patients were followed up for a mean of 31.8 months (4–71) post-metalwork removal. Data from the second operation (removal) was not combined with data from the first operation (fixation).

**Table 4:** Details of metalwork removal

Detail	Mean (range)
Months from fixation to removal (range)	13.3 (5–27)
Litres blood loss < 0.1, 0.1, 0.2, 0.3 (%)	30, 1, 0, 1 (93.8, 3.1, 3.1)
Mean (range) length of surgery in minutes	69.5 (30–120)
Number (%) patients with complications	3/33 (9.1)^*^
Mean (range) length of stay in days	1.3 (0–4)
Mean (range) months follow-up from metalwork removal	31.8 (4–71)

^*^2 haematomas, 1 superficial wound infection

*Summary*: Metalwork removal is associated with a low rate of complications (1.7%) in the review. Similarly, our study had a low complication rate.

## Discussion

The systematic review highlights that metalwork removal of percutaneously placed metalwork is safe and beneficial with improvements in pain and disability scores without compromising stability and causing significant complications. The key finding from our case–control study is that removal of metalwork benefits an earlier return to work or baseline function, independent of demographics, co-morbidities, or injury level. We also found that a greater number of fixed levels were predictive of higher analgesic use at follow-up. There is reasonable agreement between the findings of the systematic review and our case–control study in terms of timing of surgery, pain outcomes, and complication rates. However, in our study, the ODI measures were not significantly different between groups and we did not objectively assess deformity measures. Deficient in the systematic review were return to work rates and operative factors of metalwork removal (blood loss, length of stay), warranting further studies.

Patients often mention that the fixation limits their range of movements, causes stiffness, and causes intermittent pain (may be related to occupation) that is positional or related to prominent metalwork under the skin. Theories of instrumentation-related symptoms include micromotion, metal fretting, allergic reaction, low-grade infection, and stress concentration at the adjacent segments [6, 9, 10, 19, 22]. These factors likely influence return to work or baseline activities but may not be captured by the ODI or pain score and are eliminated by metalwork removal.

Our systematic review revealed that although the question of whether percutaneous screws should be retained or removed has received little attention, the published reports favour removal. Most studies report that metalwork removal improves pain [2, 4, 8, 11, 13, 14, 20] and, importantly, there are no reports of worse pain. There are also significant improvements in disability or the chance of returning to work [2, 8, 14, 20, 21]. Removing the metalwork does not cause progressive deformity in most patients [2, 11, 13, 16, 20, 21]. The increased deformity reported in a few patients [8] is clinically unimportant, because it was associated with improved pain scores and did not necessitate additional surgery. The complication rate is low, and the complications are minor and short term. On average, most surgeons remove the metalwork after a year [2, 8, 11, 13, 14, 16, 20, 21], although there is evidence that earlier removal may be associated with an increased range of movements [20].

### Study limitations

The limitations of published studies include heterogeneity of outcomes and patient inclusion criteria, no class I evidence, and no controls. There is no specific consideration to operative factors of metalwork removal surgery. Limitations of our case–control study include the small numbers of patients, retrospective nature, loss to follow-up, and lack of long-term radiological stability data. Due to the small numbers of patients, it was impossible to examine the effect of patient age or length of construct on outcome. However, we used demographically matched groups to compare outcomes, and none of our cohort with the metalwork removed required further surgery for kyphotic deformity correction, which is in line with the literature. None of the studies (including ours) performed an economic analysis to compare the cost of removal surgery versus the financial gains of improved quality of life and return to work after metalwork removal. In addition, none of the studies (including ours) evaluated the psychosocial morbidity, which may influence subjective measures of postoperative pain and return to normal function after spinal injury.

Based on the literature review and our case–control study, we make the following eight recommendations to guide spinal surgeons facing the question of whether to remove the metalwork after percutaneous fixation of thoracolumbar fractures. We note that there is no class I evidence, and the strength of recommendations (SOR) is based on a small number of studies with small numbers of patients:

1. Should the metalwork be removed? The review and our study favour metalwork removal. SOR: moderate.
2. Does it make a difference whether there is neurological deficit or not? From the review, both patient groups may benefit. SOR: moderate.
3. Does metalwork removal reduce pain? There is evidence of improvement in pain; however, this finding is not consistent across studies. SOR: weak.
4. Does metalwork removal reduce disability? There is evidence of improvement in disability; however, this finding is not consistent across studies. SOR: weak.
5. Does metalwork removal improve the chances of returning to work? Yes, based on our study, not the review. SOR: weak.
6. Will metalwork removal de-stabilize the spine to require further surgery? There is consistent evidence that the spine does not de-stabilize and that removal does not lead to further surgery. SOR: strong.
7. What is the optimal time for metalwork removal? On average, one year after insertion. There is evidence that earlier removal (6–12 months) is equally safe and effective and more likely to restore movement. SOR: moderate.
8. What does the removal surgery involve? Based on our experience, it involves a small operation under general anaesthesia. Hospital stay is usually 1–2 nights. The risk of complications is low, and the commonest complications are minor, including superficial wound infection and hematoma. SOR: strong.

## Conclusions

Based on the systematic review and our study, metalwork removal is safe and may confer benefits including reduced pain and disability and increased chance of returning to work. On average, removal was performed approximately 1 year after the injury. The level of evidence is low.

## Supplementary Information

Below is the link to the electronic supplementary material.

Supplementary file1 (DOCX 25 KB)

## Data Availability

The datasets generated and analysed during the current study are available from the corresponding author on reasonable request.

## Abbreviations

ASIA: American Spinal Injury Association Impairment Scale
AVHR: Anterior vertebral height ratio
CI: Confidence interval
CA: Cobb’s angle
CL: Correction loss
C%: Compression percentage
CSI: Canal stenosis index
DA: Deformation angle
DD: Degree displacement
FA: Fracture angle
KA: Correction loss
L: Lumbar
LOS: Length of stay
NRS-B/L: Numeric Rating Scales for Back and Leg pain
ODI: Oswestry Disability Index
PICO: Population, Intervention, Comparison, Outcome [framework]
ROM: Range of movement
RTA: Road traffic accident
RTW: Return to work
SF-12: 12-Item short-form survey
SI: Sagittal index
SMA: Segmental motion angle
T: Thoracic
TLICS: Thoracolumbar Injury Classification and Severity Scale
TLJ: Thoracolumbar junction
VAS: Visual Analog Scale
VBH: Vertebral body height
VH: Vertebral height loss
