# Supplementary material for: Removal or retention of minimally invasive screws in thoracolumbar fractures? Systematic review and case–control study
Source: Acta Neurochir (Wien). 2023 Feb 15;165(4):885–95. doi: 10.1007/s00701-023-05514-9 (PMC10068661; doi:10.1007/s00701-023-05514-9)
Supplement: Supplementary file 1 — Supplementary file1 (DOCX 25 KB) [file 701_2023_5514_MOESM1_ESM.docx]

**Supplemental Table 1.** Binary logistic regression. Dependent variable is taking analgesics (Yes=1, No=0).

| **Predictor** | **Values** | **Odds ratio** | **95% C.I.** | **P value** |
| --- | --- | --- | --- | --- |
| *Univariate* | | | | |
| Screws removed | Yes=1, No=0 | 3.394 | 0.834 – 13.810 | 0.088 |
| Age | Years | 1.010 | 0.971 – 1.052 | 0.614 |
| Charlson | Comorbidity | 1.094 | 0.598 – 2.000 | 0.771 |
| Injury level | T=1, TLJ=2, L=3 | 0.899 | 0.355 – 2.277 | 0.823 |
| Vertebra | Number of levels | 2.123 | 1.104 – 4.082 | 0.024^*^ |
| *Multivariate* | | | | |
| Screws removed | Yes=1, No=0 | 2.594 | 0.601 – 11.195 | 0.201 |
| Vertebra | Number of levels | 1.940 | 1.010 – 3.788 | 0.043^*^ |

*C.I., confidence interval; L, lumbar; T, thoracic; TLJ, thoraco-lumbar junction*

*Predictors with P<0.1 in univariate analysis were used in multivariate model*

^*^*Significant difference*

**Supplemental Table 2.** Multinomial logistic regression. Dependent variable is ODI (0-20%=0 mild, 21-40%=1 moderate, 41-60%=2 severe, 61-80%=3 crippled, 81-100%= 4 bed bound).

| **Predictor** | **Values** | **Nagelkerke R^2^** | **Chi^2^** | **P value** |
| --- | --- | --- | --- | --- |
| *Univariate* | | | | |
| Screws removed | Yes=1, No=0 | 0.081 | 4.634 | 0.201 |
| Age | Years | 0.006 | 0.305 | 0.959 |
| Charlson | Comorbidity index | 0.016 | 0.893 | 0.827 |
| Injury level | T=1, TLJ=2, L=3 | 0.018 | 0.973 | 0.808 |
| Vertebra | Number of levels | 0.099 | 5.755 | 0.124 |

*L, lumbar; T, thoracic; TLJ, thoraco-lumbar junction*
